# Impact of philanthropic investment on integrating social determinants of health into diabetes care at US federally qualified health centers

**DOI:** 10.1016/j.dialog.2025.100248

**Published:** 2025-10-08

**Authors:** Sonak D. Pastakia, Alycia Clark, Katie Lewis, Damon Taugher, Omolola Adeoye-Olatunde, Kourtney Byrd, Kay Johnson, Andrew M. Gonzales, Nader Tossoun, Danielle Cortez, Alejandra Mata, Christy Ward, Pua Akana, Rachel Randall, Rina Ramirez

**Affiliations:** a Purdue University Center for Health Excellence Quality and Innovation, Fifth Third Bank Building, 640 Eskenazi Ave, Indianapolis, IN 46205, USA; b Direct Relief, 6100 Wallace Becknell Road, Santa Barbara, CA 93117, USA; c Indy Healthnet, 3403 E. Raymond St, Indianapolis, IN 46203, USA; d Northeast Valley Health Corporation, 1172 N. Maclay Avenue, San Fernando, CA 91340, USA; e Share Our Selves, 27725 Santa Margarita Pkwy, Mission Viejo, CA 92691, USA; f Wahiawa Health, 302 California Ave, Wahiawa, HI 96786, USA; g Zufall Health, 18 West Blackwell Street, Dover, NJ 07801, USA

**Keywords:** Diabetes, Federally qualified health centers, Philanthropic foundations, Social determinants of health

## Abstract

**Study objective:**

Assess the impact of a change in a philanthropic funding strategy toward focusing on the inclusion of responses to the social determinants of health (SDOH) in diabetes care.

**Design:**

Retrospective analysis of routinely collected clinical and social determinants of health data.

**Setting:**

Federally Qualified Health Centers Across the United States who were selected to receive funding after applying.

**Participants:**

People living with diabetes who received care support that was partially or wholly supported from philanthropic funding provided by Direct Relief.

**Interventions:**

The primary intervention was the injection of funding from Direct Relief to support the integration of interventions responsive to the SDOH. Example interventions include referral to SDOH support, home based monitoring, inclusion of community health workers, virtual care, and community-based care.

**Main outcome measures:**

The primary outcome measure was the change in HbA1c from baseline to two to four months for all patients contributing data.

**Results:**

Participants in the HBHC program demonstrated a reduction in glycosylated hemoglobin of −1.25 points ([95 % CI, −1.45 - -1.06], *p < 0.01*) after 60-to-119-days. Participants with family and housing needs, nutrition needs, and social and emotional health needs had statistically significantly higher baseline HbA1c's than patients without these needs.

**Conclusion:**

Future philanthropically supported efforts should encourage integration of SDOH interventions into clinical services for under-resourced patients living with diabetes. Additional prospective, controlled studies should be completed to more definitively determine the impact of investment on specific interventions designed to respond to the most frequently encountered SDOH needs.

## Highlights

• Philanthropic funders rarely track the impact of their investment on health outcomes.
• Prescriptive approaches that focus only on clinical needs are typically used.
• Direct Relief changed to a holistic approach that responded to social determinants.
• Significant reductions in the baseline HbA1c were observed within 60 to 119 days.
• Patients with certain social determinant needs had higher baseline HbA1c's.

## Graphical abstract

**Figure:**
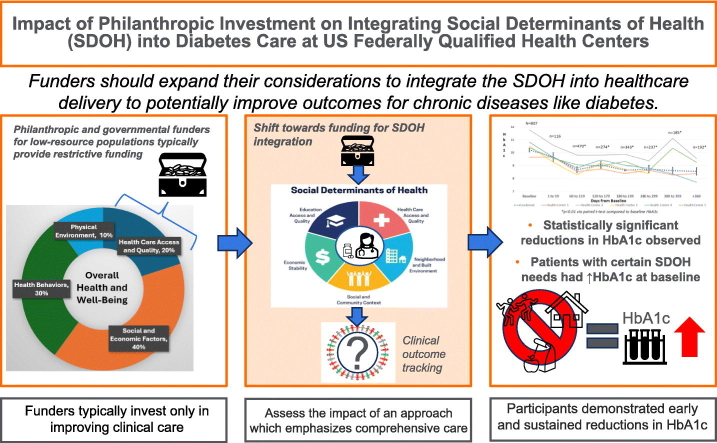

## 1 Introduction

The health care system in the United States continues to underperform in addressing the health needs of populations experiencing poverty. The lack of progress on narrowing health disparities continues to drive the poor overall performance of the US healthcare system. This lack of progress is especially true for diseases like diabetes where certain racial and ethnic minority groups and groups with lower socioeconomic status continue to have increased rates of complications with minimal progress in narrowing the gaps. [[1], [2], [3], [4]]

Because of the many challenges populations experiencing poverty face in affording and accessing health care, many rely on subsidized or free care provided by federally qualified health centers (FQHC) or heavily subsidized charitable clinics which try to respond to their many clinical needs. [5] With the growing awareness of the outsized impact of non-clinical social determinants of health (SDOH) amongst resource-constrained populations, many of these clinics are beginning to incorporate programs which respond directly to SDOH needs. [6] The SDOH are “broadly defined as the conditions in which people are born, grow, live, work and age, and people's access to power, money and resources.” Numerous agencies such as the Centers for Disease Control, Institute of Medicine, National Academies of Science, American Diabetes Association, and World Health Organization (WHO) have provided guidance on the importance of integrating SDOH-related services/interventions into clinical care. [[7], [8], [9], [10], [11], [12]] Unfortunately, only limited funding exists to support this integration and many clinics continue to struggle with funding interventions which are responsive to non-clinical SDOH needs. [4]

The COVID-19 pandemic has exacerbated many of the SDOH needs populations face, especially for people living with diabetes as they faced higher mortality from COVID-19 than almost any other population. [13] While meta-analyses of the impact of COVID-19 on glycemic control in people living with diabetes has not shown statistically significant changes overall, numerous studies have highlighted how the pandemic has disproportionately impacted underrepresented minority populations which tend to have a higher prevalence of diabetes. [[14], [15], [16], [17]] These challenges contributed to the dramatic increase in mortality for non-white Hispanic and African American populations as they experienced a drop of 3.7 years and 3.2 years of life expectancy respectively. [18,19]

Similar to many federal agencies, corporations and their philanthropic foundations have made renewed commitments to addressing societal inequities as a result of the growing awareness of the disproportionate toll of the COVID-19 pandemic on underrepresented minorities and the growing awareness of the health disparities that continue to plague US society. [20] In a survey of 200 grantmaking corporations and foundations during the height of the COVID-19 pandemic in September 2020, 82 % of the respondents noted that their programming has changed or will change with many of them trying to provide assistance to communities. Furthermore, 60 % reported creating new or expanded initiatives to address disparities. [21] Unfortunately, however, there has been only limited evaluation of the impact of donor funded SDOH interventions on clinical outcomes. [22] While COVID-19 ended as a Public Health Emergency of International Concern (PHEIC) in 2023, it remained an ongoing public health problem especially for people living with diabetes. Recent reviews have found that patients with poorly managed diabetes are at nearly double the risk of severe consequences from COVID-19 infection especially when sugars are poorly managed. [23]

With the growing awareness of the impact of SDOH related inequities on under-resourced populations, the not-for-profit humanitarian organization, Direct Relief, changed their funding approach within the Helping Build Healthy Communities (HBHC) program to encourage applicants to propose funding requests that more holistically respond to the non-clinical SDOH needs of populations experiencing poverty. This transition is in line with the goals and objectives of the WHO and the Sustainable Development Goals (SDG) as it responds to SDG 1 (poverty reduction), SDG 3 (improve health and well-being), SDG 10 (reduce inequalities), and SDG 17 (support partnerships). [24] Direct Relief established the HBHC (https://www.directrelief.org/partnership/bd/) program to support FQHCs/look-alikes in collaboration with the National Association of Community Health Centers with financial support from 10.13039/100005442BD, one of the largest global medical technology companies in the world who is *advancing the world of health™* by improving medical discovery, diagnostics and the delivery of care. This program has been operated since 2013 and has provided over 52 grants totaling $7.78 million to health centers in 20 states. After nearly a decade of managing the program, Direct Relief wanted to evaluate the impact of their funding approach which previously focused primarily on supporting clinical pharmacy activities within FQHCs/look-a-likes. Additional details and background on this program can be found in prior publications. [25,26] Over time, applicants began to focus more on diabetes because of the growing burden and expense of managing diabetes amongst populations experiencing poverty. [2] This led to a more intentional effort to evaluate the impact of HBHC on diabetes-related outcomes. This emphasis on diabetes grew during the COVID-19 pandemic as health centers tried to support their patients as health centers adopted a variety of lock-down restrictions to minimize risk. Because of these challenges, Direct Relief changed the application criteria for this program in 2020 to encourage applicants to lean into integrating responses to the SDOH needs into their efforts.

Based on the limited availability of evidence assessing the ideal investment strategies for philanthropic foundations and the role investment on interventions on SDOH needs have on diabetes related outcomes, the primary outcome this assessment sought to determine is what changes in glycemic control are observed when funders encourage integration of SDOH-related services/interventions into care. Secondary outcomes assessed include, 1) what changes in blood pressure and glycemic control are observed at different time points when SDOH-related services/interventions are integrated, 2) what changes are observed for patients with more poorly managed HbA1c's > 8.9 %, 3) how do SDOH needs impact the baseline HbA1c measurements for patients.

## 2 Materials and methods

Direct Relief received a total of 84 applications in response to the revised request for proposals which encouraged respondents to respond to the pressing SDOH challenges patients were facing during the pandemic.

Six health centers were selected for funding, and each received an unrestricted $150,000 grant from Direct Relief to implement interventions which would assist their programming for one year. These activities were designed to complement the work of their multidisciplinary teams of providers including, but not limited to physicians, clinical pharmacists, nurses, social workers, dietitians, and community health workers (CHWs). All the health centers provided clinical pharmacist supported comprehensive medication management services as part of their intervention. This service included reviews of medication therapy, creation of personal medication records, development of a medication related action plan, protocol guided care, and prescriptive authority through collaborative practice agreements in three out of the five funded health centers. Health centers were encouraged to build upon existing clinical services by incorporating responses to the pressing SDOH needs their patients were facing. This includes incorporating community health workers, dietitians, and social workers into care. Furthermore, amid the pandemic, many health centers were forced to shift to virtual care and focus their activities on diabetes related needs in part due to the heightened risk for COVID-19-related complications and the challenges with accessing care amidst the many restrictions enacted by health systems to reduce the spread of COVID-19. Grant funding was also used to support the provision of ambulatory blood pressure cuffs and glucose monitoring supplies to facilitate remote monitoring of chronic diseases at the discretion of the health centers. More detailed descriptions of the activities have been previously described elsewhere and are summarized within Table 1, Table 2. [26] Partners were given considerable flexibility to determine what mix of services to offer without restricting them to well defined and narrowly focused interventions suitable for research as the partners were more interested in assessing the real-world implications of SDOH investment on routine clinical care.

**Table 1:**
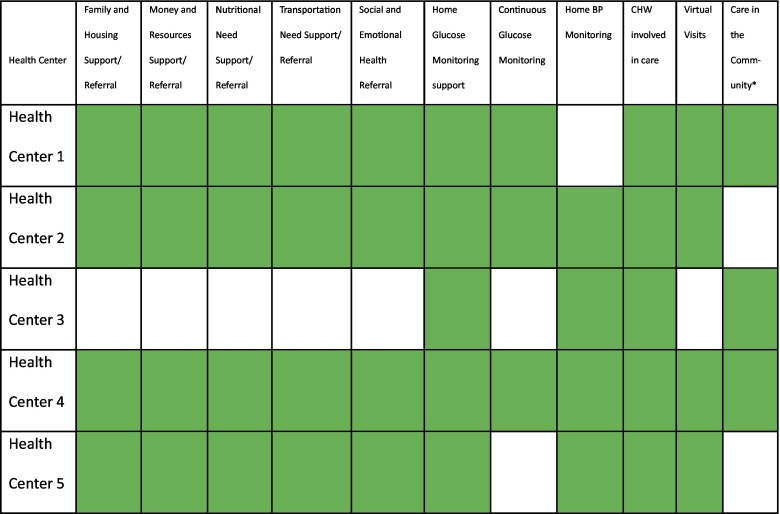
HBHC supported interventions implemented by participating health centers.

X = activity was available as part of the HBHC supported activities / O = service was not available as part of the HBHC supported activities, CHW-community health worker.

^⁎^Refers to the provision of care in locations outside of the brick-and-mortar clinical facility via mobile clinic vans, by deploying staff to meet patients at convenient locations, or provision of telehealth services.

**Table 2:** Description of the Specific Areas Partners Invested HBHC funds to Complement Ongoing Service Provision.

Site	Brief Description of the Core Activities Funded through HBHC
Health Center 1	Expand ongoing dietician services, provision of diabetes education from certified diabetes educators, and provision of pharmacist-led comprehensive medication management (CMM) to respond to the social determinants of health (SDOH) and behavioral health needs by adding a community health worker to the team, who helps connect patients to additional resources
Health Center 2	Incorporate clinical risk stratification, SDOH assessment, and social work referrals into CMM while also hiring a culturally aligned patient navigator to ensure patients receive the services and referrals, they may benefit from
Health Center 3	Enhance CMM delivered via telehealth and enhance remote monitoring by adding a culturally aligned community health worker and pharmacy technician to the care team
Health Center 4	Provide holistic CMM by including a pharmacy technician, culturally aligned community health worker, telehealth services, remote monitoring, and medication deliveries
Health Center 5	Enhance access to remote monitoring for diabetes and hypertension for high-risk low-income populations

As part of Direct Relief's renewed emphasis on monitoring and evaluation, several collaborative meetings were established with health centers to agree upon a standard template for data collection that would facilitate aggregated analysis of the data routinely obtained from the patients who received interventions supported in part by HBHC funding. After discussion, health centers agreed to provide a de-identified set of individualized data on demographic characteristics, clinician obtained clinical outcome markers (glycosylated hemoglobin and blood pressure), descriptions of who received interventions, and SDOH needs (responses to the PRAPARE questionnaire). [[27], [28], [29]] Partner meetings were scheduled every three to six months to discuss any ongoing challenges with data collection and to clarify any outstanding questions that partner clinics may have had. Upon receiving the data, errors in data entry were identified and clarified with partners as needed. In the event of unclear entries or results which did not fit the acceptable range for the different fields, those participants were excluded from the analysis. For the clinical outcome markers, health centers were asked to use a consistent method for obtaining measurements between visits regardless of whether automated/manual devices or point of care/full laboratory analyzers were utilized. In the rare event that patients relied solely on telehealth, providers educated patients on how to appropriately perform automated blood pressure checks and reported those measurements. Delays were anticipated as health centers dealt with a variety of COVID-19 related restrictions and partners were able to extend the period over which they used the funding. These delays helped to provide additional longitudinal follow-up data on patients beyond the 1 year that was originally planned. Data was submitted for evaluation after six months and 1.5 years after initiation of activities.

### 2.1 Analysis

All non-pregnant patients with type 2 diabetes who received interventions that were supported in part by HBHC funding were eligible for inclusion within the analysis. Health centers were instructed to submit data only on patients who received services that were related to HBHC funding and exclude data from other patients receiving care at their health centers. Descriptive analyses were used to describe clinic characteristics, numbers of patients, and their demographic characteristics. For the primary analysis, data from patients living with diabetes was included in the analysis if they had a baseline and at least one follow-up HbA1c recorded >59 days after the baseline measurement. They also had to have at least one reading that was >6.9 %. The primary outcome of interest was whether there were statistically significant changes in the combined HbA1c measurements of each individual patient across all funded health centers between baseline and 60-to-119-days via the paired *t*-test with a *p <* 0.05 being deemed statistically significant. In the secondary analysis, all other time points where HbA1c data was collected were compared to baseline with a *p <* 0.05 being deemed statistically significant. We also completed secondary analyses on the changes in glycemic control observed for patients with more poorly managed diabetes with baseline HbA1c levels >8.9 % at different time points throughout the funding period. Furthermore, the paired *t*-test was performed to determine the association of the SDOH needs on the HbA1c at baseline for patients who had the PRAPARE form completed. Descriptive analyses utilizing means and 95 % confidence intervals were used to describe the relationship of the mean HbA1c and the number of SDOH needs identified. For the analysis of blood pressure, descriptive statistics were used to describe the 95 % confidence intervals around the mean systolic and diastolic blood pressure (blood pressure) for all participants who contributed results during each of the time blocks. The paired t-test was used to compare the changes in blood pressure compared to baseline. For all analyses, means, 95 % confidence intervals, and the sample size available at each time point were reported along with the associated *p*-values.

Because of the reliance on routinely collected clinical information, patients were not expected to have clinical data in all the time intervals or have completed responses to all the PRAPARE questions. In the event of a missing response on a PRAPARE question, patients were excluded from those secondary analyses looking at HbA1c changes in the presence of specific SDOH needs.

The evaluation was approved by the Indiana University / Purdue University Indianapolis Institutional Review Board.

## 3 Results

A total of six health centers received funding from the HBHC program, however, only five provided data that was analyzable and included in this analysis. The funded health centers predominantly served underrepresented minority populations as the overall race and ethnicity breakdown of the populations served illustrated that 8.4 % were Asian, 4.4 % were Native Hawaiian/ Other Pacific Islander, 9.1 % were black/African American, 0.3 % were American Indian, 42.2 % were white Hispanic or Latino/a, 18.6 % were non-Hispanic white, 6.4 % were more than one race, and 10.7 % did not report their race. Additional details on the race and ethnicity breakdown found at each funded health center can be found in Appendix Table A.1. [30]

Health centers used HBHC funding to support a combination of interventions which responded to the clinical and non-clinical SDOH related needs as seen in Table 1, Table 2.

**Fig. 1:**
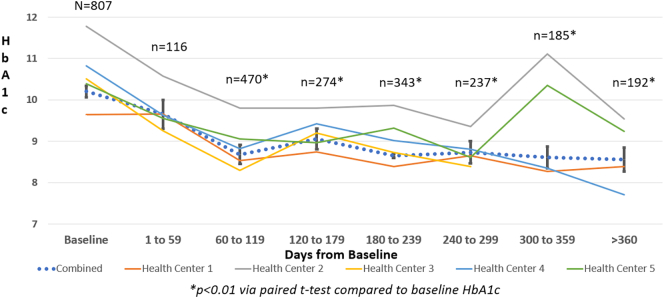
Trends of Changes in HbA1c from Baseline to >360 Days from Baseline by Each Health Center and Combined.

**Fig. 2:**
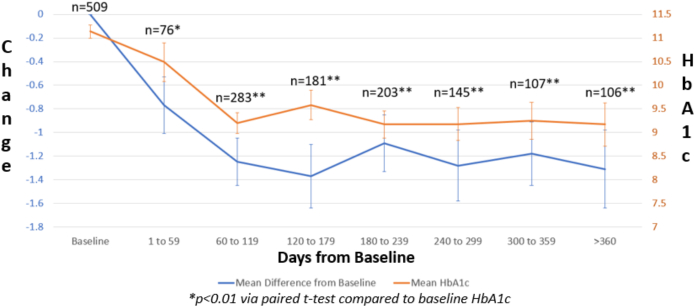
Trends in the Change in HbA1c and Mean Difference in HbA1c from Baseline to >360 Days for Patients with a Baseline HbA1c >8.9 %.

A total of 1367 patients were impacted by the funding provided by HBHC across the five supported clinics between January 2021 and June 2022. Of these, 807 patients had a baseline or follow-up HbA1c above 6.9 at some point during the evaluation. As illustrated in Fig. 1, the highest number of follow-up HbA1c's were completed between 60 and 119 days. For the primary outcome, the mean overall HbA1c during this period was statistically significantly reduced from a baseline HbA1c of 10.20 (95 % CI, 10.06–10.34) to 8.68 ([95 % CI, 8.57–8.91], *p < 0.01*) with a mean reduction of −1.25 ([95 % CI, −1.45 - -1.06], *p < 0.01).* Statistically significant reductions were sustained at all subsequent time points for both the mean HbA1c and mean reduction in HbA1c. Each of the participating health centers individually demonstrated statistically significant reductions in HbA1c between 60 and 119 days in addition to various other time points of evaluation as seen in Fig. 1. In the secondary analysis of patients with a baseline HbA1c >8.9 %, the mean overall HbA1c during this period was statistically significantly reduced from a baseline HbA1c of 11.14 ([95 % CI,11.00–11.27) to 9.20 ([8.98–9.41], *p* < 0.01) with a mean reduction of −1.88 ([−2.14–1.62], *p* < 0.01) after 60–119 days as seen in Fig. 2.

### 3.1 Intersection of SDOH and glycemic control

In the analysis of the relationship between the five main SDOH domains assessed by the PRAPARE tool and baseline HbA1c's, statistically significant elevations were noted amongst participants with family and housing needs (11.13 [95 % CI, 10.85–11.40], *p < 0.01*), nutrition needs (11.20 [95 % CI, 10.92–11.47, *p < 0.01*]), and social and emotional health needs ([95 % CI, 10.60–11.19], *p < 0.01*) as seen in Table 3. In addition, the mean baseline HbA1c in relation to the number of SDOH needs can be seen in Table 4. The small number of patients with needs in all five SDOH categories had a markedly elevated mean baseline HbA1c of 11.00 (95 %CI, 8.97–13.04).

**Table 3:** Baseline HbA1c in relation to the Social Determinants of Health for participating patients with HbA1c > 6.9.

Social Determinant of Health Need (N = number responding to the question) [#, % responding yes, #, % responding no]	**Need Identified Mean Baseline A1c** **(95 %CI) [*p*]**	**No Need Baseline A1c** **(95 %CI)**
**Family and Housing Need** **(*N* = 420)** **[yes = 153, 36.4 % no = 267, 63.6 %]**	**11.13** **(10.85–11.40)** **[<*0.01*]**	**10.03** **(9.79–10.27)**
**Nutrition Need** **(*N* = 244)** **[yes = 158, 64.8 % no = 86, 35.2 %]**	**11.20** **(10.92–11.47)** **[<*0.01*]**	**10.56** **(10.20–10.91)**
Transportation Need (*N* = 227) [yes = 61, 22.0 % no = 166, 73.1 %]	10.75 (10.27–11.23) [0.63]	10.87 (10.61–11.13)
Money and Resources Need (*N* = 246) [yes = 61, 24.8 % no = 185, 75.2 %)	10.97 (10.74–11.25) [0.92]	11.00 (10.74–11.25)
**Social and Emotional Health Need** **(*N* = 338)** **[Yes = 176, 52.1 % no = 162, 47.9 %]**	**10.90** **(10.60–11.19)** **[<0.01]**	**9.85** **(9.56–10.15)**

**Table 4:** Baseline HbA1c in relation to the Number SDOH Needs Identified.

(n = number with that number of needs, % of respondents)	**Baseline HbA1c, mean (95 % CI)**
Five SDOH Needs Identified (*n* = 5, 1.1 %)	11.00 (8.97–13.04)
Four SDOH Needs Identified (*n* = 13, 2.8 %)	9.84(9.62–10.10)
Three SDOH Needs Identified (*n* = 42, 9.1 %)	9.44 (8.70–10.20)
Two SDOH Needs Identified (*n* = 159, 34.6 %)	9.01 (8.61–9.41)
One SDOH Need Identified (*n* = 238, 51.7 %)	8.84 (8.56–9.14)
Zero SDOH Needs Identified (PRAPARE Form Completed) (*n* = 3, 0.65 %)	9.51 (8.66–10.36)

### 3.2 Blood pressure

Amongst the 1367 patients who received interventions supported by HBHC funding, statistically significant reductions in systolic blood pressure were noted with nearly every subsequent timepoint after baseline. At baseline, participants had a mean SBP of 133.2 mmHg which decreased to a mean SBP of 129.3 mmHg at 60-to-119-days (Mean Difference − 2.5, [95 % CI, −3.7 to −1.3, *p* < 0.05 via paired *t*-test]). Similar trends were noted with diastolic blood pressure; however, statistically significant reductions began during the 60-to-119-day time period (Mean Difference − 1.4, [95 % CI, −2.3 - -0.5, *p* < 0.05 via paired t-test]) and persisted through every subsequent period of evaluation.

## 4 Discussion

This analysis illustrates how resource-constrained populations relying on FQHCs/look-alikes had elevated baseline HbA1cs that were significantly improved through the integration of SDOH-related services/interventions by health centers receiving support from the HBHC program (mean difference, −1.25 ([95 % CI, −1.45 - -1.06], *p* < 0.01). This is a statistically and clinical significant reduction as any reduction >0.5 % is typically considered a relevant change in glycemic control by international guidelines and it is beyond the margin of error for point of care HbA1c lab tests. A reduction of 1.25 would result in a sizable reduction in morbidity and mortality for patients living with diabetes based on prior studies. [[31], [32], [33]]

The goals of this paper are different from the currently available literature, in that the primary interest was to assess the clinical impact that is observed when funders more broadly encourage the integration of SDOH into care rather than trying to specifically address a variety of contextualized interventions. Based on the limited availability of evidence assessing the ideal investment strategies for philanthropic foundations and the role investment on interventions on SDOH needs have on diabetes related outcomes, the primary outcome this assessment sought to determine is what changes in glycemic control are observed when funders encourage integration of SDOH-related services/interventions into care. The primary challenge with assessing each of the individual interventions is that as part of the funders' shift to this strategy, there was a desire to avoid being prescriptive with the partners in telling them what they could spend the money on or burden them with the detailed tracking requirements that would be required to assess each of their independently decided interventions. Instead of tracking the impact of specific interventions, the assessment focused on clinical outcome changes associated with this new investment strategy. [34] The findings from this study highlight that timely philanthropic investments with a more holistic view of health can help in improving diabetes control even during an unprecedented viral pandemic. These results illustrate the potential gains that can be realized as philanthropic foundations trying to address health disparities shift from focusing only on clinical needs to prioritizing a more comprehensive strategy that responds to the multi-faceted SDOH needs of resource-constrained populations. These findings mirror other studies which have highlighted that investments in SDOH can improve clinical outcomes for resource-constrained populations with diabetes. [2,35,36] Furthermore, the detailed assessment of the interplay between glycemic control and SDOH needs highlights the potential impact SDOH needs can have on compromising clinical outcomes. While many corporations operate on quarterly or annual key performance indicators, philanthropic activities designed to assist communities with SDOH related needs must adopt a longer-term approach to address the most vulnerable populations who suffer from myriad challenges with housing, nutrition, and social health.

### 4.1 Limitations

There was considerable variability between the health centers in the size of the population enrolled, the precise design of the interventions, and the completeness of data submitted. These challenges limit the ability to determine the exact impact of the different interventions utilized by partners. Fortunately, all of the interventions have been extensively studied in prior clinical trials and it is not within the scope of this paper to try to demonstrate the effectiveness of interventions which were varied between the partner clinics. [34,37,38] Future evaluations of programs like HBHC will be conducted with more longitudinal data, larger sample sizes, and greater standardization of enrollment criteria to better elucidate which interventions are most impactful and relevant for supporting resource-constrained populations.

Because of the desire to limit the intrusiveness of data collection and rely solely on retrospective, routinely collected data by the already stretched staff, there was considerable missing data and PRAPARE questionnaires were not completed for every patient. Another potential limitation is the variability in follow-up time as health centers were instructed to provide data on patients who enrolled at any time point during the funding period. This obscured the tracking of retention and loss to follow-up data as the amount of clinical outcome data each patient contributed was limited by the timing of their enrollment as patients enrolled toward the end of the funding period could only contribute a limited amount of data. This was partially addressed by requiring that each patient included in this analysis contributed at least one follow-up HbA1c. There is also the potential for selection bias where patients who benefitted from the services were more likely to stay within the program and contribute data. The lack of data also compromised the evaluation of SDOH needs as it was limited to the 460 patients who had a PRAPARE questionnaire completed. While there are several methodological limitations with the analysis, the assessment of this program is a positive step forward as it is one of the largest studies to evaluate integration of the SDOH into FQHC's across five representative regions across the US. [26] This initial evaluation will help to inform clinic leaders philanthropic funders, and eventually larger government-based funding agencies about the clinical value of adopting strategies which integrate SDOH into care for populations experiencing poverty.

### 4.2 Future steps

Based on many of the observations of this program, Direct Relief has modified the HBHC program further to provide additional investments to four of the originally funded health centers to address the identified SDOH challenges and provide longer term support for addressing the challenges observed in this analysis. Future studies will be prospective in nature, longer in duration of follow-up, and capture more detailed and complete assessments of the participants to facilitate the detection of more detailed sustained patterns in the trends amongst participants receiving various interventions. In addition, health centers and patients have been invited to participate in qualitative surveys to develop a deeper understanding of the ways in which funders can more effectively support resource-constrained populations. Future investments will be designed around the preferences of health centers and patients to continue to address the many longstanding inequities under-represented minority and resource-constrained populations face when trying to overcome SDOH related barriers.

## Data statement

Data will be furnished upon request to any interested investigators or collaborators.

## CRediT authorship contribution statement

**Sonak D. Pastakia:** Writing – review & editing, Writing – original draft, Visualization, Validation, Supervision, Resources, Methodology, Investigation, Formal analysis, Data curation, Conceptualization. **Alycia Clark:** Writing – review & editing, Writing – original draft, Validation, Resources, Methodology, Investigation, Formal analysis, Data curation, Conceptualization. **Katie Lewis:** Writing – review & editing, Supervision, Resources, Project administration, Funding acquisition, Conceptualization. **Damon Taugher:** Writing – review & editing, Supervision, Resources, Project administration, Funding acquisition, Conceptualization. **Omolola Adeoye-Olatunde:** Writing – review & editing, Validation, Formal analysis. **Kourtney Byrd:** Writing – review & editing. **Kay Johnson:** Writing – review & editing, Supervision, Project administration, Methodology, Funding acquisition, Conceptualization. **Andrew M. Gonzales:** Writing – review & editing, Supervision, Methodology, Conceptualization. **Nader Tossoun:** Writing – review & editing, Supervision, Project administration, Methodology, Funding acquisition, Conceptualization. **Danielle Cortez:** Writing – review & editing, Supervision, Project administration, Methodology, Funding acquisition, Conceptualization. **Alejandra Mata:** Writing – review & editing, Supervision, Project administration, Methodology, Funding acquisition, Conceptualization. **Christy Ward:** Writing – review & editing, Supervision, Project administration, Methodology, Funding acquisition, Conceptualization. **Pua Akana:** Writing – review & editing, Project administration, Methodology, Funding acquisition, Conceptualization. **Rachel Randall:** Writing – review & editing, Supervision, Project administration, Methodology, Funding acquisition, Conceptualization. **Rina Ramirez:** Writing – review & editing, Supervision, Project administration, Methodology, Funding acquisition, Conceptualization.

## Ethics approval

The evaluation was approved by the Indiana University / Purdue University Indianapolis Institutional Review Board.

## Funding

The work was conducted in response to grant funding received from Direct Relief which manages the Helping Build Healthy Communities (HBHC) initiative which is supported by BD.

## Declaration of competing interest

Sonak Pastakia reports financial support was provided by Direct Relief. Sonak Pastakia reports a relationship with Direct Relief that includes: consulting or advisory. Author Sonak Pastakia serves as a consultant for the philanthropic efforts of the not for profit, humanitarian assistance organization, Direct Relief, and helped organize, analyze, and describe the program in this manuscript.

If there are other authors, they declare that they have no known competing financial interests or personal relationships that could have appeared to influence the work reported in this paper.

## Figures and Tables

**Table A.1:** Overall Race and Ethnicity Breakdown of Funded Health Centers included in this Analysis.

Health Center	%Asian	% Native Hawaiian / Other Pacific Islander	%Black/ African American	% American Indian/ Alaska Native	%White Hispanic or Latino/a	%White non-Hispanic or Latino/a	% more than one race	% unreported race
Health Center 1	5.1 %	0.7 %	28.3 %	0.2 %	21.6 %	34.8 %	4.9 %	4.4 %
Health Center 2	2.2 %	0.2 %	6.3 %	0.3 %	69.2 %	12.5 %	1.9 %	7.5 %
Health Center 3	2.6 %	0.4 %	1.1 %	0.3 %	62.4 %	17.9 %	0.2 %	15.2 %
Health Center 4	29.6 %	20.4 %	3.4 %	0.0 %	1.7 %	13.1 %	20.6 %	11.1 %
Health Center 5	2.5 %	0.5 %	6.2 %	0.5 %	56.2 %	14.5 %	4.2 %	15.4 %
Mean	8.4 %	4.4 %	9.1 %	0.3 %	42.2 %	18.6 %	6.4 %	10.7 %
